# Antibiotic Susceptibility Pattern and Biofilm Formation in Clinical Isolates of* Enterococcus* spp.

**DOI:** 10.1155/2019/7854968

**Published:** 2019-03-03

**Authors:** Sridevi Shridhar, Biranthabail Dhanashree

**Affiliations:** Department of Microbiology, Kasturba Medical College, Mangalore, Manipal Academy of Higher Education, Manipal, India

## Abstract

*Enterococcus* is a commensal in the intestine and is now emerging as a drug-resistant pathogen. It produces different virulence factors.* Enterococcus* surface protein (esp) is a virulence factor that helps in the adhesion, but its role in biofilm formation is still contradictory. Moreover, in many bacterial species, strong biofilm producer exhibits multidrug resistance. Hence, this study is done to know the antimicrobial susceptibility pattern of* Enterococcus* spp. and to correlate the drug resistance with biofilm production and* esp* gene. Enterococcal isolates were collected from various clinical specimens. Antibiotic susceptibility testing was done by disc diffusion, and biofilm production was performed by microtiter plate method. PCR was performed for detection of* esp *gene. Two* E. faecium* strains resistant to vancomycin and high-level aminoglycoside (HLAR) were non-biofilm-producers and did not harbor* esp* gene. However, other biofilm-producing* E. faecium* harbored* esp* gene, and this association was found to be statistically significant (p=0.024). It was observed that there was no significant association between biofilm formation and presence of* esp* gene in* E. faecalis. *Moreover, a significant correlation was not found between drug resistance and biofilm production in both* Enterococcus* species. Thus, biofilm formation is not always associated with the presence or absence of* esp* gene and or drug resistance in* Enterococcus* spp.

## 1. Introduction

*Enterococci* are normal flora of oral cavity, gut, and female genital tract of humans and are known to cause nosocomial infections [1–4].* E. faecalis* is responsible for 80-90 percent and* E. faecium* 5-10 percent of the human enterococcal infections [5, 6]. Most frequent infections caused by* Enterococcus *spp. are urinary tract infections followed by intra-abdominal abscesses and bloodstream infections [7].

A high mortality rate of enterococcal infections is due to increasing resistance of the organism to *β*-lactam antibiotics, aminoglycosides, and glycopeptides and inadequate response to the treatment [5, 8]. Pandemic spread of vancomycin-resistant* Enterococci* (VRE) and acquisition of resistance to newer antimicrobials warrant continued surveillance and early detection of VRE along with Minimum Inhibitory Concentrations (MIC) [9].

Biofilm protects* Enterococci* from host immune response and antibiotics. Biofilm-producing* Enterococci* cause recurrent, chronic, and antibiotic-resistant infections [10–12]. According to the National Institute of Health, 80% of infections are related to biofilm-forming microbes [13, 14]. Apart from biofilm-forming ability,* Enterococcus *spp. are known to produce various virulence factors [15]. Moreover, clinical isolates have been reported to harbor gene coding for* esp* virulence factor rather than the commensal strains [16]. Hence, the study was done to know the prevalence of drug resistance in clinical isolates of* Enterococcus* spp. and to find the association of drug resistance with biofilm formation and* esp* genes in this part of the country.

## 2. Materials and Methods

### 2.1. Isolation and Identification of* Enterococcus* spp.

*Enterococci* isolated from clinical samples like pus, sputum, vaginal swab, and aspirates (n=150) received for routine culture at the Department of Microbiology, Kasturba Medical College (KMC) Mangalore, from December 2016 to June 2017 were included in the study. Institutional Ethics Committee, KMC Mangalore, India, has approved this study. All the media, antibiotic discs, and chemicals used in the study were procured from Hi-Media Laboratories Pvt Ltd., Mumbai, India. Enterococcal isolates were identified by colony characteristics and common biochemical reactions [17] and confirmed by VITEK-2 automated system (bioMérieux, USA).

### 2.2. Antibiotic Susceptibility Test

Antimicrobial susceptibility to ampicillin (10*μ*g), penicillin (10 units), tetracycline (30*μ*g), erythromycin (15*μ*g), chloramphenicol (30*μ*g), vancomycin (30*μ*g), teicoplanin (30*μ*g), ciprofloxacin (5*μ*g), and nitrofurantoin (300*μ*g) was determined by Kirby-Bauer disk diffusion [18] and interpreted as per CLSI guidelines [19]. The quality control strain used was ATCC* E. faecalis *29212.

### 2.3. High-Level Aminoglycoside Resistance (HLAR) Detection

Detection of HLAR was performed with disks containing gentamicin (120*μ*g) and streptomycin (300 *μ*g) by disk diffusion method. Results were read after incubation at 35°C for 24h and after 48h for streptomycin. A zone diameter of 6mm indicates resistance, 7-9mm shows that the results are inconclusive, and more than 10mm suggests that the isolates are sensitive to aminoglycosides. Resistance by disc diffusion to gentamicin corresponds to MIC of >500 *μ*g/ml, and susceptibility corresponds to MIC of < 500 *μ*g/ml. However, for high-level streptomycin, MIC of >1000 *μ*g/ml by broth dilution and >2000 *μ*g/ml by agar dilution method corresponds to a zone diameter of 6mm by disk diffusion. MIC of ≤500 *μ*g/ml by broth and ≤1000 *μ*g/ml by agar dilution corresponds to 10 mm diameter by disk diffusion method [19].

### 2.4. Detection of MIC for Vancomycin

Enterococcal isolates were inoculated onto Muller Hinton Agar supplemented with 5% defibrinated sheep blood. Vancomycin E-strips (Ezy MIC™) were placed on the inoculated plates and incubated at 37°C in 5% CO_2_ for 24 h. The MIC was read where the ellipse intersects the MIC scale on the strip.* E. faecalis* ATCC 29212 and ATCC 51299 were used as negative and positive controls, respectively. The results were interpreted as sensitive (MIC ≤4*μ*g/ml), intermediate (MIC 8-16 *μ*g/ml), and resistant (MIC ≥32 *μ*g/ml) based on the CLSI guidelines [19].

### 2.5. Biofilm Formation

All the clinical isolates were checked for biofilm production by the procedure used by Kafil and Mobarez (2013) and Triveda and Gomathi (2016) [20, 21]. Briefly, freshly subcultured strains of* Enterococcus* on blood agar plates were inoculated in 1ml of Brain Heart Infusion (BHI) broth with 1% glucose and incubated at 37°C for 24 h. To 180*μ*l of fresh BHI medium, 20*μ*l of 24-hour-old bacterial growth was added, which corresponded to a turbidity of 0.5 McFarland standard. 200*μ*l of the suspension of the clinical isolates and the control strain (*E. faecalis* ATCC 29212) were inoculated into flat bottom microtiter plates in duplicates and incubated at 37°C in 5% CO_2_ for 24h. After incubation, the contents of the plate were removed, tapped, and washed three times with phosphate buffer saline. The biofilm was fixed by adding 150*μ*l of methanol for 20 min. It was air-dried for about 30 min in an inverted position and later stained with 0.1% crystal violet for 15min. Excess stain was removed, and plates were washed with distilled water. 150*μ*l of 33% acetic acid was added in each well and kept for 30 min without shaking. The optical density (OD) was measured at 570nm. Based on the OD values, the isolates were categorized as strong biofilm formers (OD_570_> 2), medium (OD_570_> 1 but <2), weak (OD_570_> 0.5 but <1), and non-biofilm-formers (OD_570_≤ 0.5) [13].

### 2.6. Detection of the* esp* Gene by Polymerase Chain Reaction (PCR)

All the isolates of* E. faecalis* and* E. faecium* were subjected to PCR for the detection of the* esp* gene. DNA extraction was done by boiling method. Briefly, three to four colonies of enterococcal isolates were suspended in 100*μ*l of distilled water. The bacterial cells were lysed by boiling for 10 minutes in a dry bath. The lysate was centrifuged briefly, and 2*μ*l of the supernatant was used as the DNA. PCR was done by using primers* esp* 11 (5′- TTGCTAATGCTAGTCCACGACC-3′) and* esp* 12 (5′-GCGTCAACACTTGCATTGCCGAA-3′). Nuclease-free water and* E. faecalis* ATCC 29212 were used as* esp *negative and positive controls, respectively. PCR reaction mixture consisted of 200*μ*M of dNTP mixture and 2.5 U Taq polymerase with 1X buffer and 25mM MgCl_2_, 0.2*μ*M of primers, and 1*μ*l of DNA. The PCR tubes containing master mix, primer, and DNA were amplified in a thermocycler (Bio Rad Inc., USA). PCR reaction conditions were initial denaturation at 95°C for 2 min, followed by 30 cycles of 94°C for 45 secs, 63°C for 45 secs, and 72°C for 1 min. Final extension was carried out at 72°C for 10min. The amplified product was resolved by agarose gel electrophoresis using 1.5% agarose in 1X Tris-acetate EDTA (TAE) buffer. The gel was stained with 0.5 mg/ml ethidium bromide. Gels were visualized under UV transilluminator, and gel pictures were photographed using gel documentation system (Alpha View 1.3.0, Alpha Innotech Corporation Multi Image Light Cabinet) [15, 22].

### 2.7. Statistical Analysis

The data were tabulated and analyzed by statistical package SPSS ver11.0 (Chicago, IL, USA) to compare antibiotic resistance between different clinical isolates and biofilm production in the presence/absence of* esp* gene among* Enterococcus* spp. Chi-square test was used for categorical variables and P value < 0.05 was considered as significant.

## 3. Results

### 3.1. Distribution of Enterococcus spp.

A total of 150* Enterococcus* isolates were included in the study. Among these, 82 (58%) were* E. faecalis *and 63 (42%) were* E. faecium. *56.3% of the* E. faecalis* and 69.9% of* E. faecium* were isolated from the males, whereas 43.7% of* E. faecalis* and 30.1% of* E. faecium* were isolated from female patients.* Enterococcus* spp. isolated from different clinical samples are shown in Table 1. Antibiotic susceptibility was performed on all 150 isolates. However, biofilm and* eae* gene detection was done on 137 isolates as 13 stored isolates were lost during recovery, which included 8 (4* E. faecalis* and 4* E. faecium*) urinary isolates and 5 (4* E. faecalis* and 1* E. faecium*) isolates from pus.

### 3.2. Antibiotic Susceptibility of* Enterococcus *spp.

All the* E. faecalis* isolates were sensitive to vancomycin. Three strains of* E. faecium* were resistant to vancomycin by disk diffusion method. Among these, two isolates had a MIC of ≥32*μ*g/ml and one MIC of ≥8*μ*g/ml. Thus, based on MIC, only two strains were vancomycin-resistant. One each of vancomycin-resistant* E. faecium* isolates was from pus and tissue. Resistance pattern of* Enterococcus* spp. to various antibiotics tested is shown in Table 2. It was observed that significantly higher number of* Enterococcus *spp. isolated from tissue and pus samples showed resistance to amikacin (p=0.009), amoxiclav (p=0.002), ampicillin (p≤0.001), high-level gentamicin (p=0.004), erythromycin (p≤0.001), penicillin (p=0.006), piperacillin-tazobactam (p=0.005), and vancomycin (p=0.04) when compared to enterococcal isolates from other samples. Moreover, a significant number of urinary enterococcal isolates showed resistance to ampicillin (p=0.014) when compared to isolates from other samples. The resistance of enterococcal isolates from blood and body fluid to imipenem was found to be statistically significant (p=0.03). It was observed that significant number of* E. faecalis *from all the clinical samples showed resistance to HLG (p≤0.001) and erythromycin (p=0.009).

### 3.3. Biofilm Production

Among the 137* Enterococcus* tested for biofilm production, five (2* E. faecium* and 3* E. faecalis*) urinary isolates and one* E. faecium* from pus and one* E. faecalis* from bronchoalveolar lavage (BAL) were strong biofilm producers. Among the three strong biofilm-producing urinary* E. faecalis* isolates, one was resistant to cotrimoxazole, and one was resistant to HLG. Among the two strong biofilm-producing urinary* E. faecium* isolates, one was resistant to cotrimoxazole and HLS and another strain to ampicillin. Strong biofilm-producing* E. faecalis* from BAL was sensitive to all the antibiotics tested. Strong biofilm-producing* E. faecium* from pus was found to be resistant to HLS. However, 107 (78.1%) isolates were non-biofilm-formers which included both sensitive and resistant strains of* E. faecalis *(n=65) and* E. faecium *(n=42).

### 3.4. Detection of* esp* Gene by PCR

**Figure 1:**
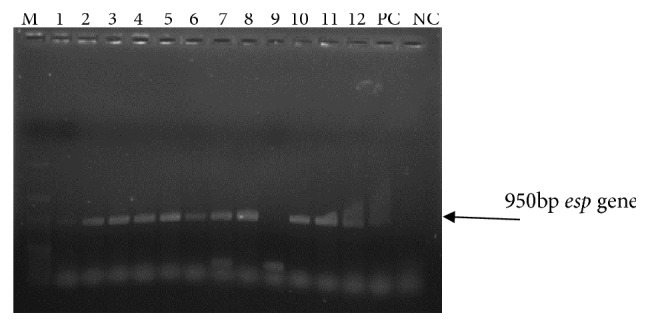
Agarose gel picture of PCR reaction showing* Enterococcus* spp. positive for* esp* gene. Lanes: M: molecular weight marker; 1: isolate negative for* esp*; 2 to 8: isolates positive for* esp*; 9: isolate negative for* esp*; 10 to 12: isolates positive for* esp*; PC: positive control for* esp*; NC: negative control for* esp*.

PCR was performed on 137 isolates for the detection of* esp* genes, and 40 isolates (22* E. faecalis* and 18* E. faecium*) were positive for* esp* gene. Agarose gel picture of PCR showing* Enterococcus* spp. positive for* esp* gene is shown in Figure 1. The relation between biofilm and presence of* esp* gene among* Enterococcus* spp. is shown in Table 3. The occurrence of* esp* gene and biofilm production was not statistically significant in case of* E. faecalis* (p =0.117), while it was statistically significant in case of* E. faecium* (p=0.024).

## 4. Discussion

*Enterococcus* is one of the significant pathogens affecting all age groups.* E. faecium* is more resistant than* E. faecalis. *Hence, speciation and antibiotic susceptibility testing are necessary to detect the emergence and changing pattern of drug resistance. Vancomycin-resistant* Enterococcus* is a significant cause of concern as this might share its resistance gene with other bacterial strains, causing crossover of gene rendering others resistant to vancomycin.

In the present study, out of 150* Enterococcus* isolates, 58% were* E. faecalis* and 42%* E. faecium*. The rate of isolation of* Enterococcus* spp. was higher from urine (46.6%) and pus (29.3%), followed by blood and body fluids (11.3%), as shown in Table 1. Earlier studies from India and abroad report different rates of isolation of* Enterococcus* spp. from clinical samples, which ranged from 10 to 80% from urine, from 16 to 43% from pus, and from 3 to 36% from blood [4, 6, 23–25]. Isolation rate of* Enterococcus* spp. in the current study is at par with few of the earlier studies [6, 26]. Thus, our report and reports from earlier workers from India and abroad clearly indicate that variation in isolation rate depends on the geographical area and the clinical samples chosen in the study.

In our study, 73.1% and 53% of* E. faecalis* isolated from urine were resistant to HLG and HLS, respectively. Meanwhile the rate of resistance of urinary* E. faecium* was found to be 48.2% to amoxiclav, 65.5% each to HLG and piperacillin, and 68.9% to HLS. (Table 2). A similar earlier study on urinary* Enterococcus* isolates from India reported resistance for HLG (40%), piperacillin (54%), nitrofurantoin (11.5%), and vancomycin (8.5%) [25]. However, in our study, two (3.1%) of* E. faecium *strains were resistant and all the* E. faecalis* strains were sensitive to vancomycin. One each vancomycin-resistant* E. faecium* strain from pus and tissue were resistant to HLAR and sensitive to teicoplanin. In an Iranian study by Talebi* et al.,* the resistance pattern was different from that of our research, where isolates were resistant to teicoplanin (3%) and vancomycin (9%), along with few other drugs [27]. This shows that resistance varies from region to region or from institution to institution in the same area. Hence, it is essential to know the antibiogram of the enterococcal isolates in an area to formulate antibiotic policy.

Resistance to erythromycin was shown by a higher number of* E. faecium* strains (p=0.002) as they are intrinsically resistant to macrolides, lincosamides, and streptogramin B (MLSB phenotype). Cross-resistance to all macrolides arises from modification of the 23S rRNA target (except linezolid resistance) by a variety of methylase genes, commonly* ermB*. Hence, macrolides and lincosamides are not used to treat enterococcal infections, even if* E. faecalis* and* E. faecium* are susceptible to quinupristin-dalfopristin in vitro [28]. In the present study, erythromycin was tested for its susceptibility just to know the resistance pattern and not to use for treatment.

In this study, 21.9% of enterococcal isolates produced biofilm, which included 27.5%* E. faecium *and 17.7%* E. faecalis*. A study from Tamil Nadu [29] showed 68% isolates to be biofilm formers. The study used isolates from diverse clinical samples and detected biofilm formation by three different methods: microtiter plate method, tube method, and Congo red method. In our study, we have used only microtiter plate method. Thus, the method used for the detection of biofilm and origin of the isolate will influence the biofilm formation.

Among the biofilm-producing* E. faecalis *(n=14) isolates, 13 were resistant to HLAR, two were resistant to teicoplanin, and one was sensitive to all the antibiotics except amikacin. Among the biofilm-producing* E. faecium* (n=16) isolates, 14 isolates were resistant to HLAR. However, all the biofilm-producing* E. faecalis* and* E. faecium* were susceptible to vancomycin. Thus, vancomycin-resistant* E. faecium* was non-biofilm-producer. In the present study, 29.2% of the isolates carried* esp* gene (Figure 1), while the rest did not. In a study done by Kafil* et al*. [20, 30], 75% of the* Enterococcus* isolates producing biofilm carried the* esp* gene. A survey by Toledo-Arana* et al*. [15] reported biofilm production by 46.5% of* esp* gene carrying* E. faecalis*. However, there are no reports from India to show a clear relation between presence of* esp* gene and biofilm production.

In our study, an association of biofilm and presence of* esp* gene as depicted in Table 3 was not significant among* E. faecalis* (p=0.117), while for* E. faecium* it was statistically significant (p=0.024). A study by Kafil* et al.* [24] showed no significant association between biofilm formation and the presence or absence of* esp* gene (p>0.05). However, studies from Iran by Kafil* et al*. [30] and Toledo-Arana [15] shows that presence of* esp* gene in urinary drug-resistant* Enterococcus *isolates led to strong biofilm formation and a firm adherence to host cells. However, in our study, only ten urinary* E. faecalis *and nine* E. faecium* were biofilm-producers, and five each had* esp* gene. Of the 19 biofilm-producing urinary* Enterococcus* spp., 14 showed HLAR resistance and two were resistant to teicoplanin. Moreover, vancomycin-resistant* E. faecium *did not harbor* esp* gene and did not produce biofilm. Thus, biofilm production, presence of esp genes, and drug resistance were not interrelated in the present study.

A study by Dale* et al*. (2015) had shown evidence of* E. faecalis *genetic determinants mediating antibiotic resistance within biofilms [30]. The same research also suggests that* E. faecalis *employs biofilm-specific mechanisms and not the simple extracellular matrix diffusion barriers to keep antibiotics away from their targets. Since our study period was only of four months' duration, we targeted for* esp* gene alone. However, further research is required to study more virulence factors and correlation of the same with biofilm production and antibiotic resistance. In conclusion, biofilm formation is not always associated with the presence of* esp* gene or drug resistance. Emergence of VRE, HLAR, and resistance to teicoplanin has left us with very few therapeutic options for enterococcal infection.

## Figures and Tables

**Table 1:** *Enterococcus* spp. isolated from different clinical samples.

Clinical specimen	*E. faecalis* N (%)	*E. faecium* N (%)	Total *Enterococcus* spp. N (%)
Tissue and pus	25 (56.8)	19 (43.1)	44 (29.3)

High vaginal swabs (HVS)	07 (63.6)	04 (36.3)	11(7.3)

Bile	03 (60)	02 (40)	05 (3.3)

Urine	41 (27.3)	29 (41.4)	70 (46.6)

Blood and body fluids	09 (52.9)	08 (47)	17 (11.3)

Respiratory specimens	02 (66.6)	01 (33.3)	03 (2)

*Total*	**87 (58)**	**63 (42)**	**150**

**Table 2:** Antibiotic resistance pattern of *E. faecalis* and *E. faecium.*

Antibiotics tested	Percentage resistance		
	*E. faecalis *	*E. faecium*	*Enterococcus *spp.
Amikacin	62.2	88.5	73.2
Amoxiclav	15.2	52.5	29.5
Ampicillin	20.7	58.7	36.6
Erythromycin	40.7	66.7	80.6
High-level gentamicin	48.3	65.1	56.6
High-level streptomycin	48.3	71.4	56.6
Imipenem	36	59.5	45.9
Meropenem	42	59.5	49.4
Nitrofurantoin	7.3	24.1	14.2
Piperacillin	39	65.5	50
Piperacillin-tazobactam	25	48.8	36.7
Teicoplanin	8	12.7	10
Vancomycin	0	3.17	1.3

**Table 3:** Relationship between biofilm and presence of *esp* gene among *Enterococcus* spp.

Type of biofilm (OD_570_)	*Enterococcus* spp. with *esp* gene		*Enterococcus *spp. without *esp* gene		Total *Enterococcus* spp. N (%)
	*E. faecalis* *N (*%)	*E. faecium* *N (*%)	*E. faecalis* N (%)	*E. faecium* N (%)	
Strong (>2)	3 (13.6)	3 (16.6)	1(1.7)	0 (0)	7 (5.1)

Medium (1 to 2)	2 (9.0)	2 (11.1)	3 (5.2)	1 (2.5)	8 (5.8)

Weak (0.5 to 1)	2 (9.0)	2 (11.1)	3 (5.2)	8 (20)	15 (10.9)

Non-biofilm-formers (≤0.5)	15 (68.1)	11 (61.1)	50 (87.7)	31 (77.5)	107 (78.1)

*Total*	**22 (16.1)**	**18 (13.1)**	**57 (41.60)**	**40 (42.3)**	**137 (100)**

## Data Availability

The datasets generated and/or analyzed during the current study are available from the corresponding author upon reasonable request.

## References

[1] Winn W. C., Allen S. D., Jande W. H., Koneman P. C. (pp. 1294-1298, 2006). *Konman’s Color Atlas and Textbook of Diagnostic Microbiology*.

[2] Murray B. E., Weinstock G. M. (1999). *Enterococci: New Aspects of An Old Organism*.

[3] Richards M. J., Edwards J. R., Culver D. H., Gaynes R. P. (2000). Nosocomial infections in combined medical-surgical intensive care units in the United States. *Infection Control and Hospital Epidemiology*.

[4] Mukherjee K., Bhattacharjee D., Chakraborti G., Chatterjee S. S. (2016). Prevalence and antibiotic susceptibility pattern of enterococcus species from various clinical samples in a tertiary care hospital in Kolkata. *International Journal of Contemporary Medical Research*.

[5] Moellering R. C. (1992). Emergence of enterococcus as a significant pathogen. *Clinical Infectious Diseases*.

[6] Sreeja S., Sreenivasa Babu P. R., Prathab A. G. (2012). The prevalence and the characterization of the enterococcus species from various clinical samples in a tertiary care hospital. *Journal of Clinical and Diagnostic Research*.

[7] Low D., Keller N., Barth A., Jones R. (2001). Clinical prevalence, antimicrobial susceptibility, and geographic resistance patterns of enterococci: results from the Sentry antimicrobial surveillance program, 1997–1999. *Clinical Infectious Diseases*.

[8] Shah L., Mulla S., Patel G. P., Rewadiwala S. (2012). Prevalence of enterococci with higher resistance level in a tertiary care hospital: a matter of concern. *National Journal of Medical Research*.

[9] Tripathi A., Shukla S. K., Singh A., Prasad K. (2013). A new approach of real time polymerase chain reaction in detection of vancomycin-resistant enterococci and its comparison with other methods. *Indian Journal of Medical Microbiology*.

[10] Costerton J. (2001). Cystic fibrosis pathogenesis and the role of biofilms in persistent infection. *Trends in Microbiology*.

[11] Moniri R., Ghasemi A., Moosavi S. G., Dastehgoli K., Rezaei M. (2013). Virulence gene’s relationship with biofilm formation and detection of aac (6’)/aph (2′′) in enterococcus faecalis isolated from patients with urinary tract infection. *Jundishapur Journal of Microbiology*.

[12] Watnick P., Kolter R. (2000). Biofilm, city of microbes. *Journal of Bacteriology*.

[13] Mohamed J. A., Huang W., Nallapareddy S. R., Teng F., Murray B. E. (2004). Influence of origin of isolates, especially endocarditis isolates, and various genes on biofilm formation by enterococcus faecalis. *Infection and Immunity*.

[14] Mohamed J. A., Huang D. B. (2007). Biofilm formation by enterococci. *Journal of Medical Microbiology*.

[15] Toledo-Arana A., Valle J., Solano C. (2001). The enterococcal surface protein, esp, is involved in enterococcus faecalis biofilm formation. *Applied and Environmental Microbiology*.

[16] Upadhyaya G. P. M., Ravikumar K. L., Umapathy B. L. (2009). Review of virulence factors of enterococcus: an emerging nosocomial pathogen. *Indian Journal of Medical Microbiology*.

[17] Collee J. G., Mackie T. J., McCartney J. E. (1996). *Mackie & McCartney Practical Medical Microbiology*.

[18] Bauer A. W., Kirby W. M., Sherris J. C., Turch M. (1966). Susceptibility testing by a standardized single disc method. *American Journal of Clinical Pathology*.

[19] Clinical and Laboratory Standards Institute (CLSI) Performance standards for antimicrobial susceptibility testing: 27th informational supplement, CLSI document M100-S27.

[20] Mobarez A., Moghadam M., Kafil H. (2013). Adhesion and virulence factor properties of Enterococci isolated from clinical samples in Iran. *Indian Journal of Pathology and Microbiology*.

[21] Triveda L., Gomathi S. (2016). Detection of biofilm formation among the clinical isolates of Enterococci: An evaluation of three different screening methods. *International Journal of Current Microbiology and Applied Sciences*.

[22] Upadhyaya G. P. M., Lingadevaru U. B., Lingegowda R. K. (2011). Comparative study among clinical and commensal isolates of Enterococcus faecalis for presence of esp gene and biofilm production. *The Journal of Infection in Developing Countries*.

[23] Salem-Bekhit M., Muharram M., Hefni H., Moussa I., Alanazy F. (2012). Prevalence and antimicrobial resistance pattern of multidrug-resistant enterococci isolated from clinical specimens. *Indian Journal of Medical Microbiology*.

[24] Kafil H. S., Mobarez A. M. (2015). Assessment of biofilm formation by enterococci isolates from urinary tract infections with different virulence profiles. *Journal of King Saud University - Science*.

[25] Srivastava P., Mehta R., Nirwan P. (2011). Prevalence and antimicrobial susceptibility of enterococcus species isolated from different clinical samples in a tertiary care hospital of North India. *National Journal of Medical Research*.

[26] Mohanty S., Jose S., Singhal R. (2005). Species prevalence and antimicrobial susceptibility of enterococci isolated in a tertiary care hospital of North India. *Southeast Asian Journal of Tropical Medicine and Public Health*.

[27] Talebi M., Asghari Moghadam N., Mamooii Z., Enayati M., Saifi M., Pourshafie M. R. (2015). Antibiotic resistance and biofilm formation of enterococcus faecalis in patient and environmental samples. *Jundishapur Journal of Microbiology*.

[28] Miller W. R., Munita J. M., Arias C. A. (2014). Mechanisms of antibiotic resistance in enterococci. *Expert Review of Anti-infective Therapy*.

[29] Sindhanai V., Avanthiga S. S., Chander V. C. S. (2016). Antibiotic susceptibility pattern of biofilm forming and biofilm non forming enterococci species. *IOSR Journal of Dental and Medical Sciences*.

[30] Kafil H. S., Mobarez A. M. (2015). Spread of enterococcal surface protein in antibiotic resistant entero-coccus faecium and enterococcus faecalis isolates from urinary tract infections. *The Open Microbiology Journal*.

